# *AVT*: Multicenter aortic vessel tree CTA dataset collection with ground truth segmentation masks

**DOI:** 10.1016/j.dib.2022.107801

**Published:** 2022-01-06

**Authors:** Lukas Radl, Yuan Jin, Antonio Pepe, Jianning Li, Christina Gsaxner, Fen-hua Zhao, Jan Egger

**Affiliations:** a Graz University of Technology (TU Graz), Graz, Styria, Austria; b Computer Algorithms for Medicine Laboratory (Café Lab), Graz, Styria, Austria; c Research Center for Connected Healthcare Big Data, ZhejiangLab, Hangzhou, Zhejiang, 311121 China; d Medical University of Graz (MedUni Graz), Graz, Styria, Austria; e Institute for AI in Medicine (IKIM), University Hospital Essen (UKE), Ruhrgebiet, Essen, Germany; f Department of Radiology, Affiliated Dongyang Hospital of Wenzhou Medical University, Dongyang, Zhejiang, 322100 China

**Keywords:** Aorta, Vessel tree, CTA, Aortic dissection, Abdominal aortic aneurysm, Segmentations, Masks, Ground truth, Deep learning

## Abstract

In this article, we present a multicenter aortic vessel tree database collection, containing 56 aortas and their branches. The datasets have been acquired with computed tomography angiography (CTA) scans and each scan covers the ascending aorta, the aortic arch and its branches into the head/neck area, the thoracic aorta, the abdominal aorta and the lower abdominal aorta with the iliac arteries branching into the legs. For each scan, the collection provides a semi-automatically generated segmentation mask of the aortic vessel tree (ground truth). The scans come from three different collections and various hospitals, having various resolutions, which enables studying the geometry/shape variabilities of human aortas and its branches from different geographic locations. Furthermore, creating a robust statistical model of the shape of human aortic vessel trees, which can be used for various tasks such as the development of fully-automatic segmentation algorithms for new, unseen aortic vessel tree cases, e.g. by training deep learning-based approaches. Hence, the collection can serve as an evaluation set for automatic aortic vessel tree segmentation algorithms.

## Specification Table

**Table:** 

Subject	Information
Specific subject area	Computer Vision and Pattern Recognition
Type of data	Image
How data were acquired	The aortas are segmented from full body (neck to legs) computed tomography angiography (CTA) scans using semi-automatic segmentation techniques.
Data format	Raw
Parameters for data collection	The selection of files from the dataset collections was based on the image quality (e.g., slice thickness, contrast agent, scanning protocol), and that they include the whole aortic vessel tree.
Description of data collection	The datasets include 56 CTA scans from aortas, covering the aortic arch and its branches and the abdominal aortas with the iliac arteries. Furthermore, we include segmentations of the aortas and its branches (aortic vessel trees) as binary mask images.
Data source location	KiTS [1], [2], RIDER [3], Dongyang Hospital
Data accessibility	The datasets can be downloaded from FigShare [16]: https://doi.org/10.6084/m9.figshare.14806362
Related research articles	Yuan Jin, Antonio Pepe, Jianning Li, Christina Gsaxner, Jan Egger. title: Deep learning and particle filter-based aortic dissection vessel tree segmentation. SPIE Medical Imaging, Proceedings Volume 11600, Medical Imaging 2021: Biomedical Applications in Molecular, Structural, and Functional Imaging; 116001W (2021). DOI: https://doi.org/10.1117/12.2588220. reference: [4]

## Value of the Data

• The healthy aortas from the collection and its branches can be used to create an atlas or a statistical shape model (SSM) [5] of the aortic vessel tree, to study the geometry variability of human aortic vessel trees, etc.
• The aortic vessel trees together with the corresponding semi-automatically generated segmentations can serve as an evaluation set for automatic aortic vessel tree segmentation algorithms [4], [6].
• Researchers can use the scans and corresponding segmentations in order to train deep learning algorithms [7].
• Researchers can use the collection as basis for data augmentation [8] to increase the collection.
• The aortic vessel tree masks can easily be converted to *.stl* files, which are then 3D printable and can be used for educational purposes.

## 1 Data Description

**Fig. 1:**
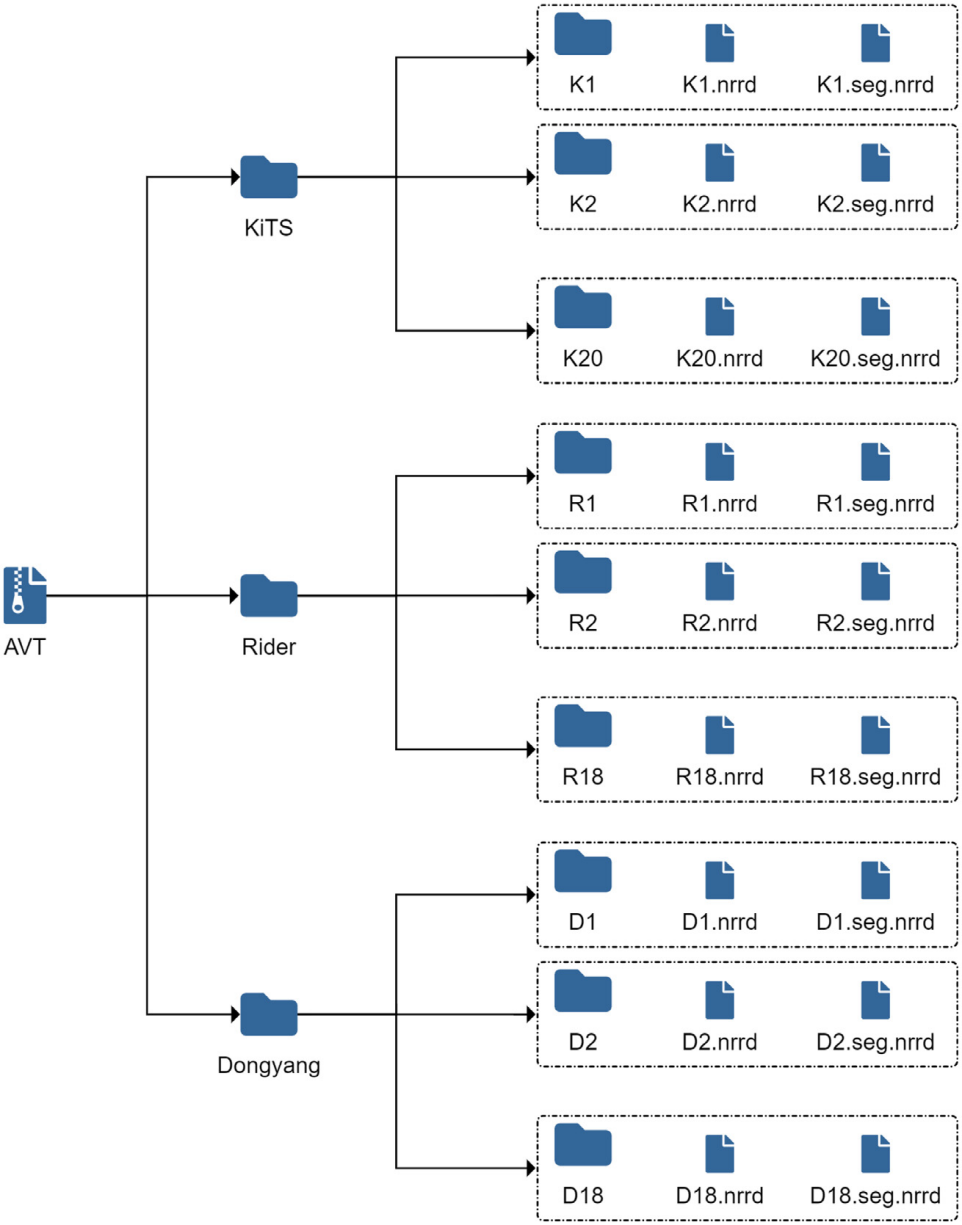
Folder structure of the *AVT* dataset collection.

In Fig. 1, we see the structure of the *AVT* dataset. The dataset includes 56 files, with a resolution of 512×{512,666}×Z, where Z denotes the number of axial slices. For each case, we provide the volume and the corresponding segmentation. Note that segmentations are saved using the composite *.seg.nrrd* file extension. This saves additional metadata and ensures 3D Slicer [9] recognizes these files as segmentations. We also use this file extension to distinguish volumes, which have the file extension *.nrrd* and segmentations.

**Fig. 2:**
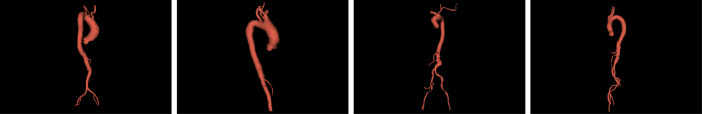
Screenshots of several healthy aorta segmentation masks of our collection from various views.

**Fig. 3:**
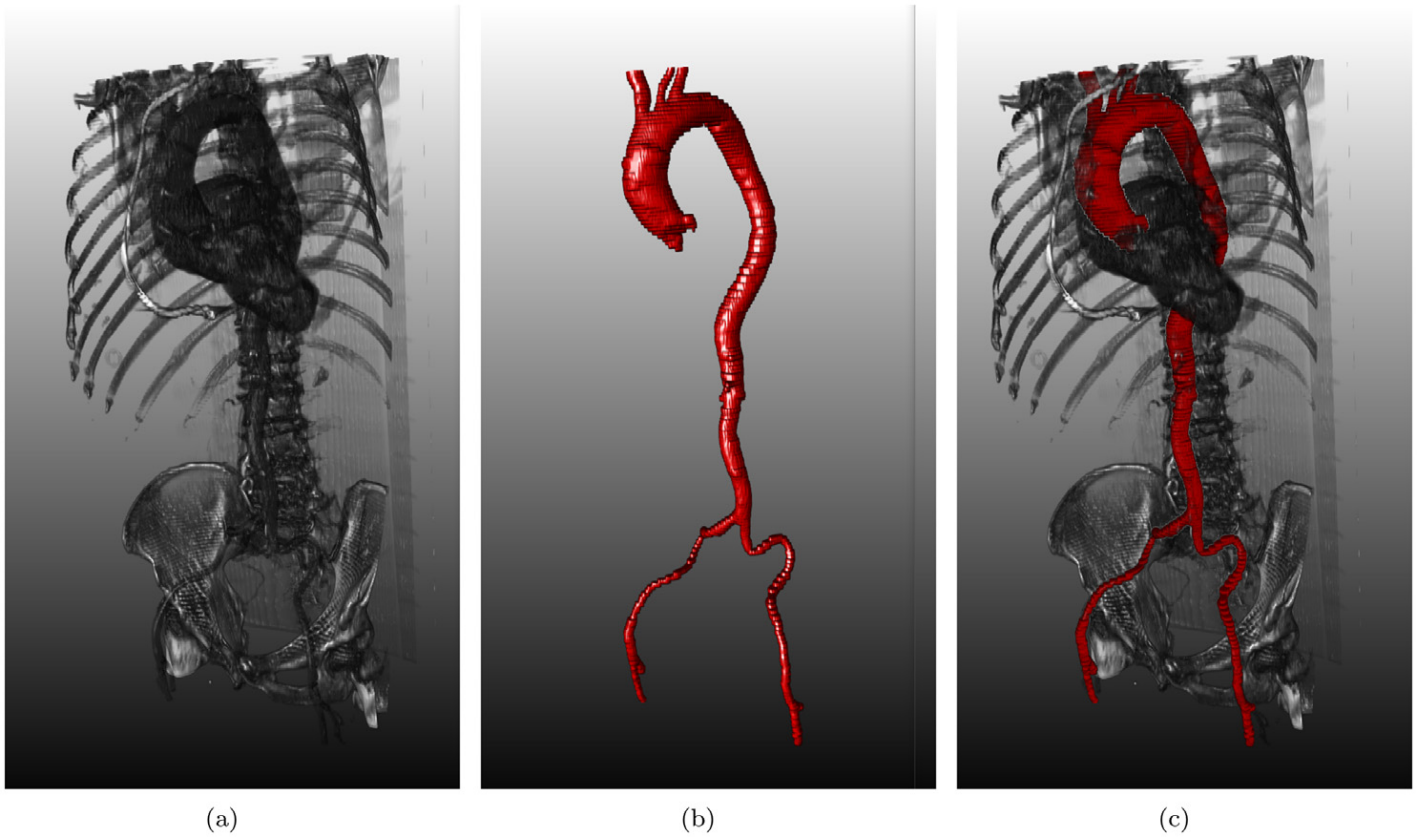
(a) Screenshot of a CTA scan of the collection. (b) Corresponding vessel tree segmentation mask. (c) Superimposed visualization of the original CTA scan and the segmentation mask.

All cases include the ascending aorta, the aortic arch, the brachiocephalic, the left common carotid, the left subclavian artery, the thoracic aorta, the abdominal aorta and the iliac arteries. Due to the variance of quality in the dataset, other branches such as the celiac trunk or the superior mesenteric artery are only visible in cases with a larger amount of axial slices. In Fig. 2, we see some example segementations from our dataset. If the number of axial slices was low, some branches were not visible in the CT scan. Therefore, for some cases, not all branches are present in the segmentations. In Fig. 3, we show a segmentation embedded in the surrounding anatomy. Our segmentation exhibits different pathologies, such as aortic dissections (AD) [10] or abdominal aortic aneurysms (AAA) [11]. In the RIDER folder, we have one case with AAA and five cases with ADs, whose directories are marked.

## 2 Experimental Design, Materials and Methods

The *AVT* dataset was constructed based on full-body CTA scans, which were taken from the KiTS19 Grand Challenge [1], [2], the Rider Lung CT dataset [3] and cases from the Dongyang Hospital. Originally, the files were in the Nearly Raw Raster Data (*nrrd*) format [12]. In Table 1, we provide statistics for an overview of our dataset, such as resolution, number of axial slices, etc. The total segmentation time for all 56 cases adds up to 58.33 hours.

**Table 1:** Image information of the aortic vessel trees. Number of axial slices, segmentation times, slice thickness and vessel tree volume are given as: min/median/max.

Image Information	KiTS	RIDER	Dongyang
x/y**resolution**	512×512	512×512	512×666
**Axial slices**	94/146/1059	260/1008/1140	122/149/251
**Slice thickness**	0.5/5/5 mm	0.625/0.625/2.5 mm	2/3/3 mm
**Pathologies**	None	AD, AAA	None
**Vessel Tree Volume**	55.8/284.5/464.4 ml	176.6/354.1/614.0 ml	126.0/254.9/488.1 ml
**Segmentation Times**	30/118/30.5 min.	27/422/80 min.	12/35/19.5 min.
**Number of Cases**	20	18	18

### 2.1 Computer-aided aortic vessel tree segmentation masks generation

The semi-automatic segmentations of the aortic vessel trees have been done using 3D Slicer (https://www.slicer.org/) [9]. The overall workflow starts with selecting and loading an aortic CTA (.nrrd file) into Slicer. Afterwards, we remove noise as a pre-processing step for the segmentation. We choose gradient anisotropic diffusion, for its capabilities in edge-preservance and selected the parameters as shown in Table 2.

**Table 2:** Parameters for gradient anisotropic diffusion. Lower conductance will preserve edges better.

Case Description	Conductance	Iterations	Time Step
Little Noise/High Resolution	0.85	1	0.0625
Little Noise/Low Resolution	0.8	1	0.0625
Aortic Dissection	0.7	1	0.0625

**Fig. 4:**
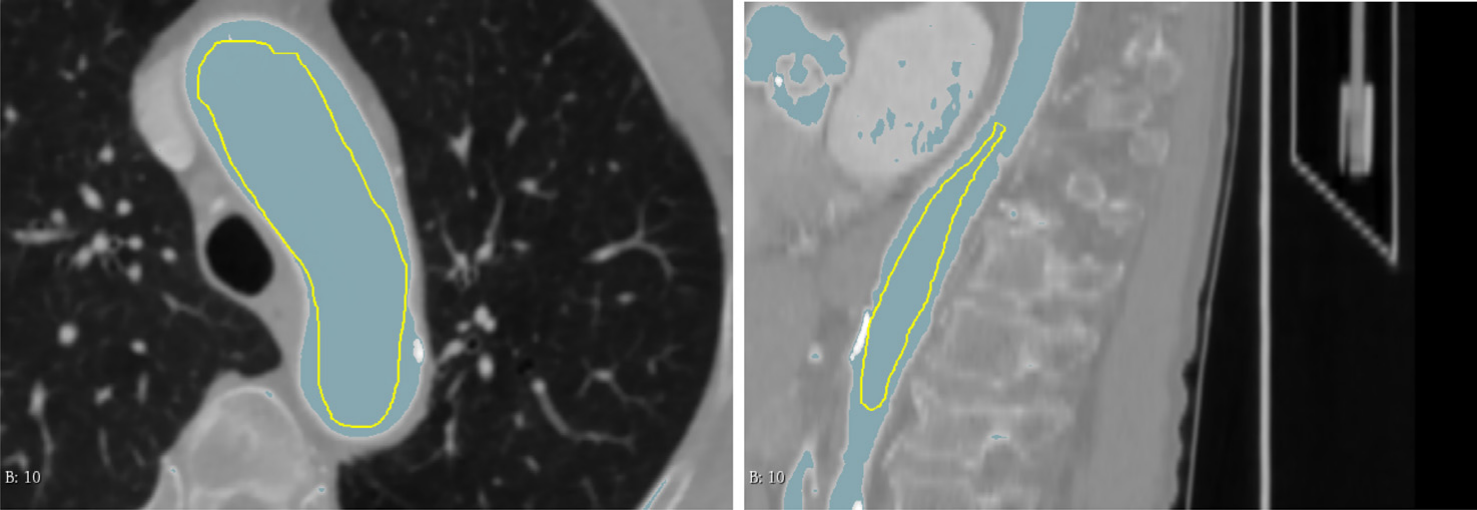
The aortic arch in the axial plane and the thoracic aorta in the sagittal plane were particularly well suited for masking in our collection. A light blue tone indicates regions to be part of the segmentation.

Next, we performed local thresholding. Therefore, we manually specified a threshold range via masking. It is important that structures that do not belong to the aorta are outside the specified threshold range. We used a minimum diameter of 3.00mm and select *GrowCut* as our segmentation algorithm. In Fig. 4, we show examples for masking of the aorta.

**Fig. 5:**
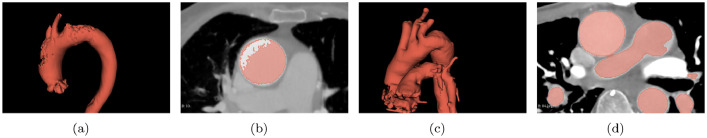
(a, b): Holes in the segmentation caused by a local threshold in 3D (a) and axial view (b). (c, d): Inclusion of the truncus pulmonalis in the segmentation in 3D (c) and axial view (d).

As we can see in Fig. 5, local thresholding alone is not sufficient for an aorta segmentation. Due to threshold differences and noise in the data, artefacts remained that needed to be corrected manually. Therefore, we used manual post-processing such as paint and erase. Using these effects, we were able to remove unwanted segments or fill in missing voxels in the segmentation. Morphological operations such as opening or closing are also of use for removing extrusion and filling holes in the segmentations. However, these effects had to be used carefully, as they might accidentally remove or connect vessels.

**Fig. 6:**
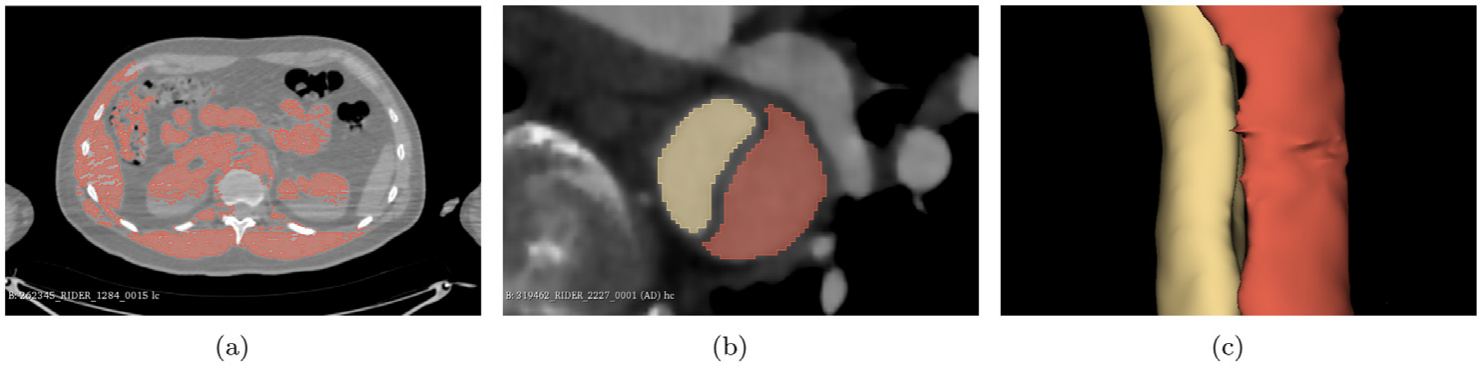
(a): Due to the quality of the scan, local thresholding returned an unsatisfactory result, thus manual segmentation was the preferred method here. (b, c): For AD cases, we created multiple segments and later combine them into one aortic mask.

The workflow specified above worked well for most cases, but same cases required a more careful post-processing or even a complete manual segmentation. In the case of aortic dissections, it might be of use to generate several segmentations (e.g. one for the true and one for the false lumen) and combine them with logical operators. As we can see in Fig. 6, some cases, like ones exhibiting AD, need a specific segmentation strategy.

AD cases can benefit, for example, from a divide and conquer approach by combining small segments to a complete segmentation of the aorta. To ensure segmentations are not connected, we can use hollow and logical operations. For cases with AD, we selected one of the two segments, as shown in Figs. 6(b) and 6(c), and created a new segmentation as a copy. Then, we applied the hollow operation to this copy, using the current segment as *inside surface*. A shell thickness of 2.5mm worked well for the AD cases in our dataset [13]. This ensures that i) multiple segments are not erroneously connected and, ii) the dissection flap is not labelled as luminal volume [14].

For some cases, such as the one in Fig. 6(a), we performed a pure manual segmentation. An option to reduce the manual work, at least for some areas of the aorta, is by segmenting only every third axial slice manually, and then using the closing operation to get the segmentation of the missing slices (in between). This worked fine for the thoracic and abdominal aorta as well as the iliac arteries. However, the aortic arch required more manual post-processing using this approach. In addition, paint and erase are needed to refine the aorta after using closing.

The segmentation process ends by exporting the segmented aortic vessel tree as 3D mask *(.seg.nrrd)* file.

## Ethics Statement

The datasets, described in the article are adapted from public collections, which are licensed under **CC BY-NC-SA 4.0** and End User License Agreement (EULA). According to the usage notes provided in their website, we make the adapted datasets public under the same licenses as the original collections.

## CRediT authorship contribution statement

**Lukas Radl:** Data curation, Writing – original draft. **Yuan Jin:** Data curation, Writing – original draft, Supervision. **Antonio Pepe:** Data curation, Writing – original draft, Supervision. **Jianning Li:** Supervision. **Christina Gsaxner:** Supervision. **Fen-hua Zhao:** Data curation, Supervision. **Jan Egger:** Writing – original draft, Supervision.

## Declaration of Competing Interest

The authors declare that they have no known competing financial interests or personal relationships that could have appeared to influence the work reported in this paper.
